# Plant–microbe networks in soil are weakened by century‐long use of inorganic fertilizers

**DOI:** 10.1111/1751-7915.13487

**Published:** 2019-09-19

**Authors:** Ruilin Huang, Steve P. McGrath, Penny R. Hirsch, Ian M. Clark, Jonathan Storkey, Liyou Wu, Jizhong Zhou, Yuting Liang

**Affiliations:** ^1^ State Key Laboratory of Soil and Sustainable Agriculture Institute of Soil Science Chinese Academy of Sciences Nanjing 210008 China; ^2^ University of Chinese Academy of Sciences No. 19A Yuquan Road Beijing 100049 China; ^3^ Rothamsted Research Harpenden Hertfordshire AL5 2JQ UK; ^4^ Institute for Environmental Genomics Department of Microbiology & Plant Biology School of Civil Engineering and Environmental Sciences University of Oklahoma Norman OK 73019 USA; ^5^ State Key Joint Laboratory of Environment Simulation and Pollution Control School of Environment Tsinghua University Beijing 100084 China

## Abstract

Understanding the changes in plant–microbe interactions is critically important for predicting ecosystem functioning in response to human‐induced environmental changes such as nitrogen (N) addition. In this study, the effects of a century‐long fertilization treatment (> 150 years) on the networks between plants and soil microbial functional communities, detected by GeoChip, in grassland were determined in the Park Grass Experiment at Rothamsted Research, UK. Our results showed that plants and soil microbes have a consistent response to long‐term fertilization—both richness and diversity of plants and soil microbes are significantly decreased, as well as microbial functional genes involved in soil carbon (C), nitrogen (N) and phosphorus (P) cycling. The network‐based analyses showed that long‐term fertilization decreased the complexity of networks between plant and microbial functional communities in terms of node numbers, connectivity, network density and the clustering coefficient. Similarly, within the soil microbial community, the strength of microbial associations was also weakened in response to long‐term fertilization. Mantel path analysis showed that soil C and N contents were the main factors affecting the network between plants and microbes. Our results indicate that century‐long fertilization weakens the plant–microbe networks, which is important in improving our understanding of grassland ecosystem functions and stability under long‐term agriculture management.

## Introduction

One of the key factors limiting the productivity of terrestrial ecosystems is nitrogen (N) availability (Vitouse and Howarth, 1991). N fertilizers are often applied to increase initial crop productivity under long‐term agricultural production. However, the long‐term application of N fertilizers may affect the plant–soil–microbe system by changing the composition and structure of plant and soil microbial communities. These effects include reductions in the richness and diversity of plant communities (Suding *et al*., 2005; Hautier *et al*., 2009), changes in the biomass and diversity of soil microbial communities (Shen *et al*., 2010; Lin *et al*., 2012) and shifts in plant–microbe interactions.

Long‐term fertilization increases the availability of nutrients such as N and phosphorus (P) in soil. These changes may alter the competition among plants for resources, such as belowground nutrients to aboveground light sources (Lamb *et al*., 2009). It was shown that in nutrient‐rich soil with adequate water content, diversity loss was mainly driven by the competition of plants for light sources (Harpole and Tilman, 2007; Hautier *et al*., 2009). Moreover, changes in soil pH under long‐term fertilization also affect species richness of plants and soil microbes. Crawley *et al*. (2005) reported that plant richness was significantly lower in the plots where soils were strongly acidified by long‐term application of ammonium sulfate compared with control or organic fertilization (147 years). The relative dominance of fungi and bacteria, as indicated by the fungal: bacterial ratios, also responds to pH. The fungal: bacterial ratio increased 50‐fold from high pH (7.4) to low pH (3.3) in the Park Grass Experiment with 150 years’ fertilization (Rousk *et al*., 2011). In addition, fertilization may also alter the life‐history strategy of soil microbes, resulting in an increase in relative abundance of copiotrophic microbes, such as rapidly growing β‐proteobacteria and Bacteroides, and a decrease in relative abundance of oligotrophic microbes such as *Acidobacteria* (Fierer *et al*., 2007, 2012).

Long‐term application of inorganic fertilizers may weaken the linkage between plants and soil microbes by reducing the dependence of microbes on plant‐derived C. Through a combination of ^13^C stable isotope detection and high‐throughput sequencing, Ai *et al*. (2015) reported that 32 years’ fertilization reduced the dependence of rhizospheric Actinobacteria on root‐derived C. Moreover, Lambers *et al*. (2009) indicated that the linkage between plants and soil microbes can be altered by changing symbiotic relationships between arbuscular mycorrhizal fungi (AMF) and plants. The application of N fertilizers can also influence the species richness and diversity of AMF by improving soil nutrient status, thus affecting their connections (Egerton‐Warburton *et al*., 2007). However, most studies are focused on the effects of fertilization over years to decades on plant–microbe relationships (Lin *et al*., 2012; Ai *et al*., 2015; Ling *et al*., 2016; Wang *et al*., 2017a,b). Fertilization for longer periods may have different effects on the contents of nutrients in soil. For example, all forms of soil P are enriched in soil that has received fertilization for over 100 years (Crews and Brookes, 2014). Consequently, the changes in microbial and plant relationships induced by chemical fertilization may also be related to the duration of fertilization. There is currently a lack of long‐term studies lasting more than a century, which can represent permanent and stable, rather than transient, responses of soil microbe–plant interactions to fertilization.

Network inference techniques are increasingly employed to decipher the relationships among microbes (Faust *et al*., 2015). These techniques range from simple pairwise Pearson or Spearman correlation measures, to more complex multiple regression and Gaussian graphical models (Zhou *et al*., 2010; Van den Bergh *et al*., 2012; Layeghifard *et al*., 2017). The application of network theory to microbiome studies can be used to model the co‐occurrence of microorganisms, find microbial relationships essential for community assembly or stability and deduce the influence of various interactions on the host health (Layeghifard *et al*., 2017). Long‐term inorganic fertilization can increase the availability of soil nutrients and thus weaken the stability of the microbial network structure; for example, microbial networks in soils with applications of N‐P‐K fertilizer become sparse and divergent (Li *et al*., 2017; Wang *et al*., 2017a,b). By contrast, a tight belowground network was found to be associated with enhanced efficiency of C uptake during semi‐natural restoration without fertilization of abandoned arable land towards a more natural system (Morriën *et al*., 2017). Therefore, changes in soil C and nutrient contents can significantly affect the soil microbial ecological network. However, most network inference studies have focused mainly on the influence of fertilization on the ecological network of soil microbial communities and there is a lack of studies of plant–microbe networks under long‐term fertilization.

The Park Grass Experiment, begun by John B. Lawes and Joseph H. Gilbert in 1856, at Rothamsted in Hertfordshire, UK, is the oldest and longest running ecological experiment in the world (Storkey *et al*., 2016). It provides a unique opportunity for studying the responses of the networks of plants and soil microbial functional communities to long‐term inorganic fertilization. In previous study, we found that long‐term fertilization altered the spatial scaling (species–area relationships) of microbial biodiversity (Liang *et al*., 2015). In the present study, we investigated soil microbes and plant networks in fertilized and unfertilized treatments to address the following questions: (i) How does fertilization for 150 years affect the networks between plant and soil microbial communities? (ii) What are the main factors affecting the networks between plants and soil microbes under fertilization?

## Results

### Variation of the major soil geochemical properties and plant/microbial community diversity

The effects of long‐term fertilization on the major soil geochemical properties (soil pH, moisture, TN, TC, NO_3_^−^–N and NH_4_^+^–N) were measured. Overall, long‐term fertilization significantly altered the major soil geochemical properties compared with the control plot (*P *<* *0.05) (Table 1). For instance, soil TN, TC and NO_3_^−^ contents increased by 58%, 82% and 82% respectively.

**Table 1:** Effects of long‐term fertilization on major soil geochemical properties

	Control	Fertilized	*T*	*P*
pH	5.14 ± 0.33	5.43 ± 0.38	−2.69	0.01
Moisture (%)	14.43 ± 0.86	15.37 ± 1.49	−2.50	0.016
TN (%)	0.31 ± 0.02	0.49 ± 0.04	−19.23	< 0.001
TC (%)	4.22 ± 0.37	7.68 ± 0.71	−19.87	< 0.001
C:N	13.67 ± 0.66	15.55 ± 0.77	−8.74	< 0.001
NO_3_^−^–N (mg kg^−1^)	2.11 ± 0.18	3.84 ± 0.35	−19.87	< 0.001
NH_4_^+^–N (mg kg^−1^)	6.83 ± 0.33	7.77 ± 0.39	−8.74	< 0.001

The significance is based on Student's *t*‐test. All data are presented as mean ± SD.

**Figure 1:**
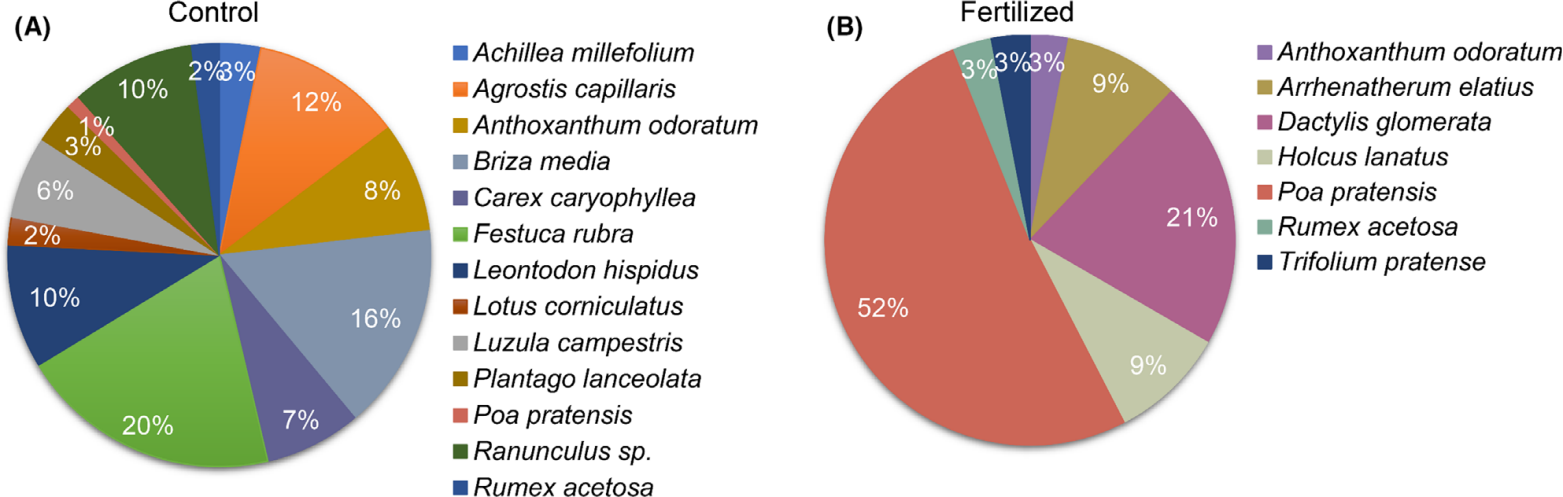
The relative frequency of plant species in (A) control and (B) long‐term fertilized plots of Park Grass.

Long‐term fertilization treatment significantly decreased the richness and diversity of plants compared with the control plot (*P *<* *0.001) (Fig. S2 and Table S1). The richness of soil microbes also significantly decreased (*P *=* *0.002). In addition, long‐term fertilization showed a tendency towards a decreased bacteria‐to‐fungi ratio (*P *=* *0.055; Fig. S3). More than 150 years of continuous fertilization distinctly altered the plant species composition and structure compared with the control (Fig. 1). For instance, we found 19 plant species in the control plot, but only 7 plant species were found in the fertilized plot. Furthermore, *Festuca rubra*,* Briza media* and *Agrostis capillaris* accounted for 44% of the total number of records in the control plot, but none of the three plants were found in the fertilized plot. In the fertilized plot, *Poa pratensis* was the dominant species (52%). These results indicate that long‐term fertilization shifts the taxonomic and functional composition of plant communities from a more diverse, stress‐tolerant community to a community with fewer species dominated by species able to compete for light.

### Effect of fertilization on microbial functional genes

**Figure 2:**
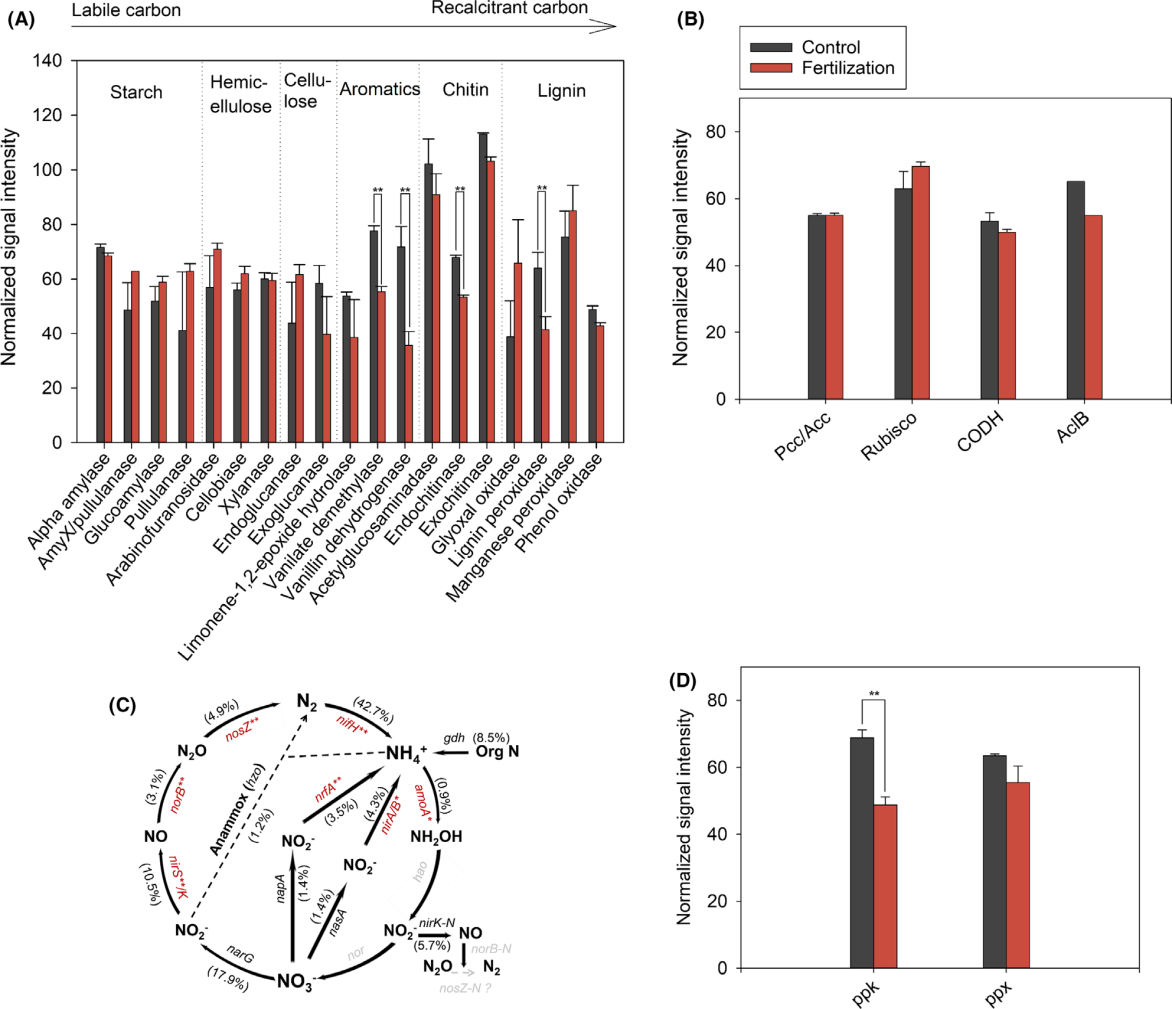
The normalized signal intensity of the detected key genes families involved (A) C degradation, (B) C fixation, (C) N cycling and (D) P cycling. The normalized signal intensities showed by cylindrical diagrams of (A), (B) and (D) were the sum of the average of individual gene sequences among 21 soil samples, divided by the number of detected sequences and then divided by 100. All data are presented as mean ± SD.*P < 0.05, **P < 0.01. The relative changes of the detected genes involved in N cycling (C) in fertilized plot were calculated, where the percentage of a functional gene in a bracket was the sum of the signal intensity of all detected sequences of this gene divided by the grand sum of the signal intensity of the detected N cycling genes, and weighted by the fold change of the signal intensity of this gene in the fertilized plot compared to that in control plot. For each functional N cycling gene, red colour means that this gene had a lower signal intensity in the fertilized plot than the control plot with significance at *P *< 0.05(*). Grey‐coloured genes were not targeted by this GeoChip, or not detected in those samples.

Microbial functional genes responsible for C fixation, C degradation, N fixation, ammoxidation, nitrification, denitrification and P utilization were detected in all samples. Long‐term application of fertilization significantly reduced the signal intensity of most genes involved in the degradation of recalcitrant C compounds (aromatics, chitin and lignin), N fixation, denitrification and P utilization (*P *<* *0.01) (Figs. 2 and S4). However, the abundance of genes involved in C fixation, ammonia oxidation and nitrification was not significantly changed by long‐term fertilization.

### Effect of long‐term fertilization on internal network of microbial communities

**Figure 3:**
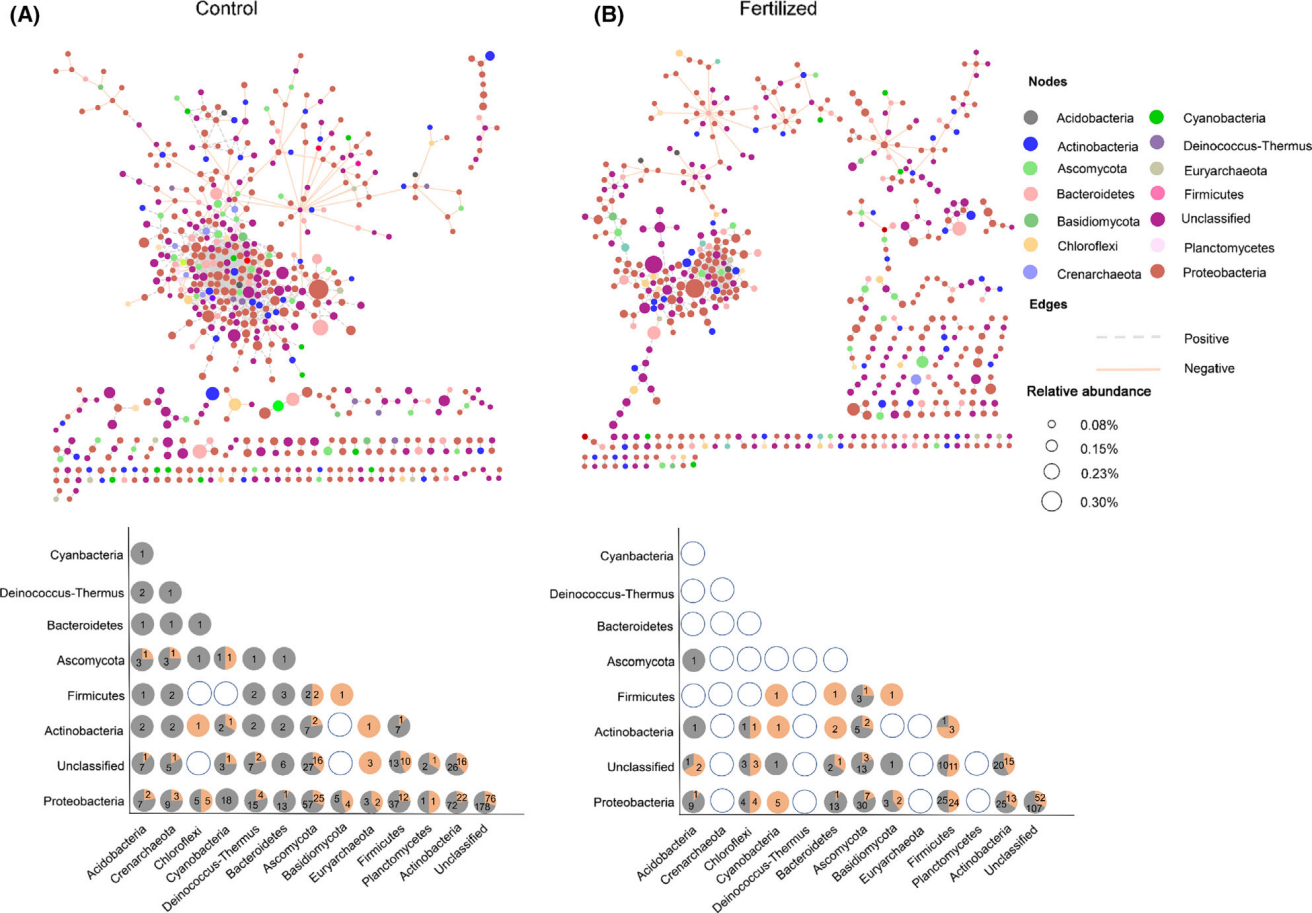
Network interactions between microbial functional communities in (A) control and (B) long‐term fertilized plots. The node size represents the relative abundance of the functional gene. Dotted grey dashed lines represent positive correlations, and orange solid lines represent negative correlations. The relationships between microbes at the phylum level were also calculated and showed below the network charts. The hollow circle represents no significant association; grey and orange in the circles represent positive and negative correlations, and numbers in the grey and orange circles represent the number of significant correlation edges.

The CoNet tool was used to determine the complex network relationships among the belowground functional microbial communities. In the fertilized plot, the distribution of microbial functional genes was sparser and more discrete compared with the control (Fig. 3, Table 2). While the number of nodes was similar in the control and fertilized networks (576 and 520 nodes in control plot and fertilized plot, respectively), the number of edges was distinctly different. The result may indicate that although the number of taxa involved in the network was similar, their potential interactions were altered. Both positive and negative associations decreased with long‐term fertilization. Furthermore, the network density and clustering coefficient of the control plot were higher than those of fertilized plot. In addition, networks among soil microbes were constructed on two spatial scales (Fig. S5 and Table S2). The results indicated that long‐term fertilization distinctly weakened the linkages among soil microbes within areas of 1 m^2^ or 5 m^2^. These results indicate that long‐term fertilization may decrease the connections among microbial functional communities.

**Table 2:** Networks properties of intrarelationship among microbial communities and inter‐relationship between plants and microbial communities in the control and long‐term fertilized plots

Networks	Plot	Number of nodes	Number of edges			Network density	Clustering coefficient
			Total	Positive	Negative		
Microbes	Control	576	1121	814	307	0.007	0.143
	Fertilized	520	672	446	226	0.005	0.116
Plant–microbes	Control	49	58	5	53	0.046	0.061
	Fertilized	15	14	0	14	0.133	0.003

### Changes in the network between plants and microbial functional genes

**Figure 4:**
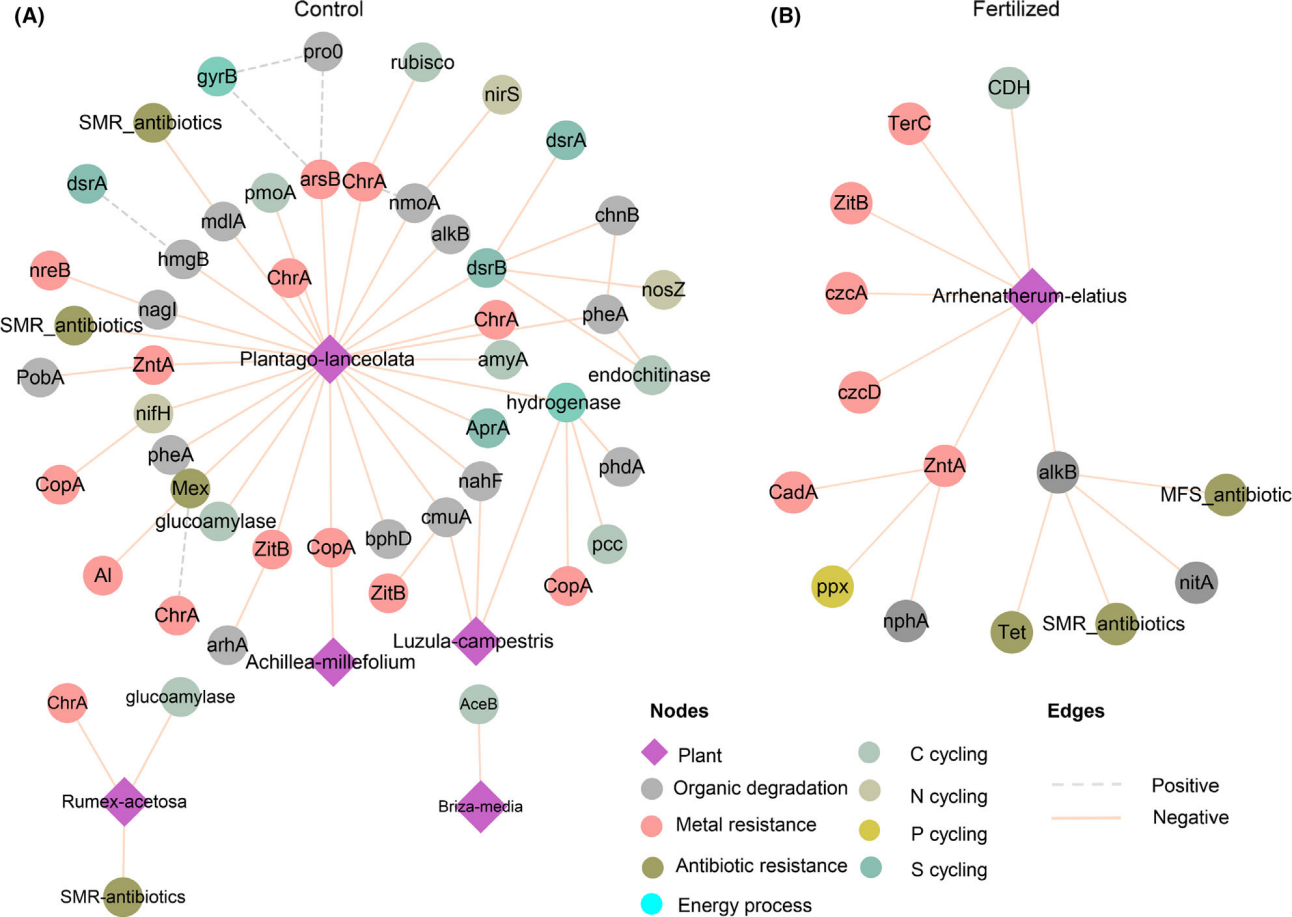
Network interactions between plants and soil microbes in (A) control plot and (B) long‐term fertilized plot. Circles and diamonds represent functional genes and plants respectively.

There are also complex ecological networks between plants and belowground microbes. The plant–microbe network was distinctly different under control and fertilized conditions (Fig. 4). For instance, in the control plot, we found that five plant species (*Plantago lanceolata*,* Luzula campestris*,* Achillea millefolium*,* Rumex acetosa* and *Briza media*) and microbial functional groups formed interaction networks, but only one plant species (*Arrhenatherum elatius*) showed interactions with the microbial functional groups in the fertilized plot. Moreover, most of the microbial groups closely linked to plants in the control plot were those functioning in C cycling and degradation. Long‐term fertilization decreased the number of these functional groups that closely linked to the plant compared with the control. The properties of networks between plants and microbial functional groups showed that fertilization decreased the number of nodes and edges, and the clustering coefficient of the network (Table 2). Additionally, this shows that more connections between plants and microbial functional genes were present in the control plot than that in the long‐term fertilized plot (Figs. S6 and S7). These results further suggested that long‐term application of fertilizers may influence the interaction between plants and functional microbial communities.

### Effects of biotic and abiotic attributes on the interaction between plants and soil functional microbial communities

Pearson's tests were performed to determine whether pH, moisture, TN, TC, C:N, NO_3_^−^, NH_4_^+^ and plant diversity affected the diversity of soil microbial functional genes (Table S3). Plant diversity was highly associated with microbial functional gene diversity (*P *<* *0.05). Of all the soil geochemical attributes, TN, TC, C:N, NO_3_^−^ and NH_4_^+^ were significantly negatively correlated with both plant and soil microbial community diversity (*P *<* *0.05). However, soil pH was only negatively correlated with plant diversity (*P *<* *0.05).

**Figure 5:**
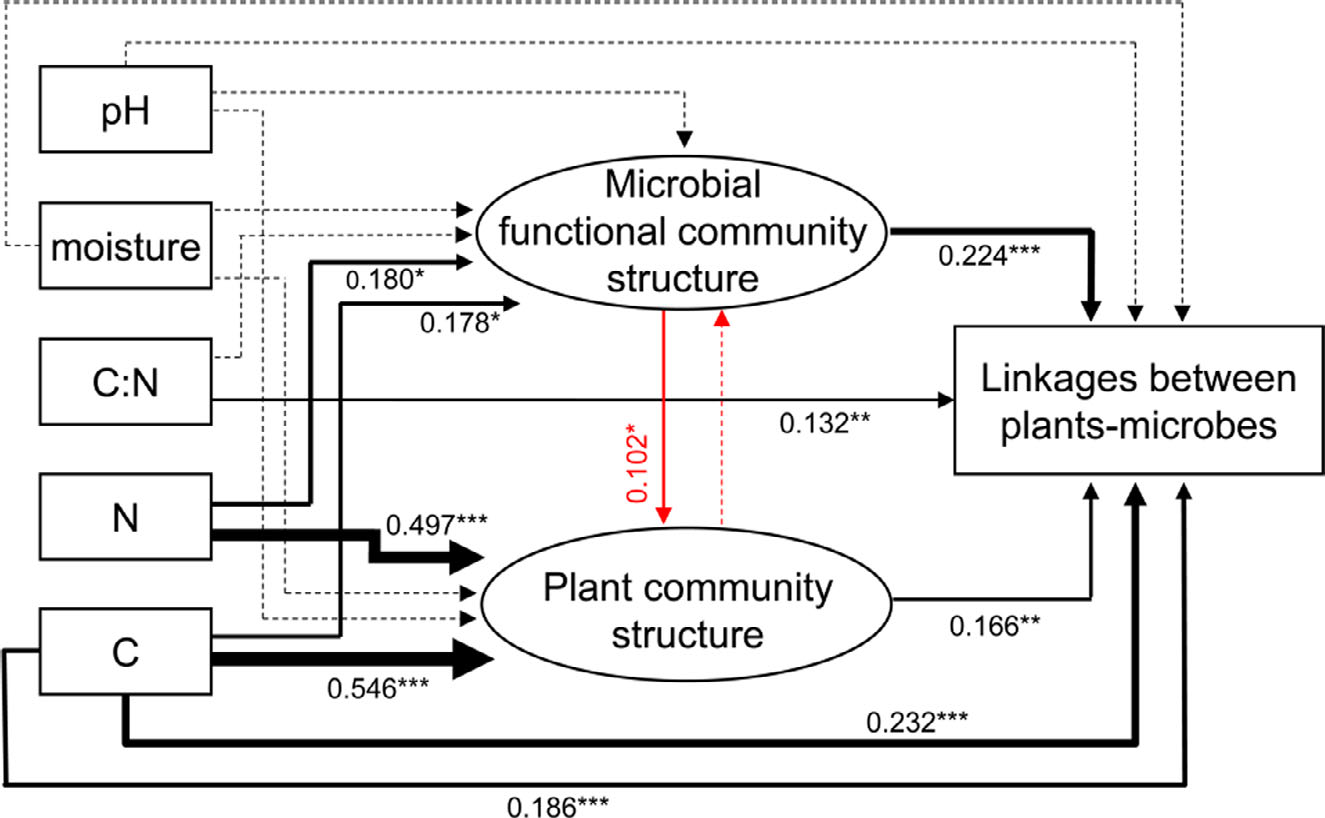
Mantel path analysis links associations between plants and microbes to soil geochemical attributes and community structure of soil microbes and plants with long‐term fertilization. Solid and dashed lines indicate significant and insignificant relations respectively; lines width is proportional to *r* the correlation coefficient. The symbols *, ** and *^**^ represent *P* < 0.05, *P* < 0.01 and *P *< 0.001 respectively.

Mantel path analysis was used to construct the association among soil geochemical properties, community structures of plants and microbial functional genes and the interaction between plants and microbes (Fig. 5). Our results showed that soil C (*r *=* *0.232, *P *=* *0.001) and N contents (*r *=* *0.186, *P *=* *0.001) were the main factors affecting the interaction between plants and microbes. Soil C and N also directly affected the community structures of plant and microbes. Soil microbial functional communities were found to significantly affect plant structure (*r *=* *0.102, *P *=* *0.023).

## Discussion

### Changes in soil C and N contents alter plant–microbe network

Consistent with the previous studies (Jiao *et al*., 2014; Wang *et al*., 2017a,b), our results showed that changes in soil C and N contents were the main factors that affect the plant–microbe networks. From the standpoint of soil C content, soil microbes such as bacteria and fungi mainly rely on the large amounts of sugars, amino acids and organic acids deposited in the plant rhizosphere (Bais *et al*., 2006). Plants may secrete a series of chemical components such as flavonoids, strigolactones or terpenoids in root exudates that can help plants recognize and select beneficial organisms from large community of pathogenic and beneficial microbes (Bais *et al*., 2006; Venturi and Fuqua, 2013; Massalha *et al*., 2017). Recruitment of beneficial microbes may be crucial for plant growth and development under environmental stress conditions such as drought, nutrient limitation, pathogen attack, pests, high salinity or heavy metal stress (Jacoby *et al*., 2017). However, the increase of soil C content over time caused by long‐term fertilization and the continuous supply of N may weaken the reliance of plants for beneficial microbes. It has been demonstrated that long‐term fertilization can reduce the dependence of microbes on root‐derived C and significantly increase their preference for soil organic matter (Ai *et al*., 2015). In the long‐term fertilized soils, this change in soil microbes may reduce the plants’ selective effect on beneficial microbes and weaken plant–microbe associations, especially those with positive ecological connections, potentially reducing resilience to stress.

Long‐term fertilization may also reduce the number of microbial C cycling genes (Fig. 2). Significantly lower abundance of microbial functional genes encoding for recalcitrant C degradation was observed, such as vanillate demethylase, endochitinase and lignin peroxidase in fertilized soils. This may reduce the turnover rate of soil organic matter because changes in functional gene abundance are related to the activity of microbial extracellular enzymes (Wallenstein *et al*., 2010; Cenini *et al*., 2016). For example, Trivedi *et al*. (2016) found a significant positive correlation between the abundance of microbial functional genes involved in C degradation and their corresponding enzyme activity in soil. High abundance of functional genes indicated that the microbes may have a higher degradation potential for recalcitrant C in the control plot. Also, we found that the overall signal intensity of genes for the utilization of recalcitrant C rather than labile C was negatively correlated to soil TC (Fig. S8, *P *<* *0.05). This result further suggested that microbes may be more proficient at degrading recalcitrant C sources in the control plot than the fertilized plot. Therefore, long‐term fertilization likely affected the connections between plants and microbial functional communities by indirectly altering the degradation rate of recalcitrant C. C partitioning between plants and microbes and soil C and N coupling can be changed with long‐term N fertilization (Giardina *et al*., 2004; Fornara and Tilman, 2012), thus affecting plant–microbe networks. It should be noted that in this study, the detection of functional genes detected by Geochip does not necessarily mean that these genes were being expressed at the time of sampling; thus, further studies should focus on transcriptomics at the RNA level.

In natural systems, N is usually bound in organic molecules and is therefore minimally bioavailable for plants (Jacoby *et al*., 2017). To access N nutrients, plants are dependent on the growth of soil microbes such as bacteria and fungi, which possess the metabolic machinery to depolymerize and mineralize organic forms of N (Bonkowski, 2004; Richardson *et al*., 2009). However, the long‐term application of inorganic N fertilizers significantly increased the bioavailability of soil N (Table 1), thereby potentially reducing the positive ecological interactions between plants and microbes. Moreover, fertilization may also reduce the heterogeneity of nutrient availability, allowing a small number of species that can adapt to high nutrient levels to spread and dominate. We propose the following inferences: (i) the small number of species that can adapt to high nutrient levels can increase interactions, such as competition, among species in the short term reducing diversity, (ii) in the long term, the reduced heterogeneity of nutrient availability will lead to a decrease in the number of plant species that can adapt to different nutrient levels, significantly decreasing the richness and diversity of plants, bacteria and fungi, thereby weakening the interconnections between plants and microbes.

### Changes in plant and microbial community structures influence plant–microbe interactions

Long‐term application of N fertilizers can reduce the richness and diversity of plants and soil bacteria and fungi (Suding *et al*., 2005; Hautier *et al*., 2009; Shen *et al*., 2010; Lin *et al*., 2012). We confirmed that changes in plant and microbial communities are likely to be important factors that reduce plant–microbe interactions for the following reasons: First, plants can release their border cells and exudates, together termed ‘rhizodeposition’ (Dennis *et al*., 2010). The amount of rhizodeposition ranges from 5% to 30% of the total plant fixed C (Bekku *et al*., 1997; Hütsch *et al*., 2002; Dennis *et al*., 2010). Rhizodeposition is essential for the growth of microbes; thus, soils with different plant species differed in the composition and abundance of microbial communities (Griffiths *et al*., 1992; Bardgett *et al*., 1999). Research has shown that there is a significant positive correlation between plant diversity and the type and amount of root exudates (Eisenhauer *et al*., 2017). Lower plant diversity will reduce the diversity and abundance of root exudates, so potentially weakening the interaction between plants and microbes. Moreover, Benizri and Amiaud (2005) have shown that floristic composition and carbon substrate utilization patterns of rhizobacterial communities were less diverse in fertilized than unfertilized plot in grassland, accompanied with a change in C and N distribution during the vegetation cycle, which probably induced changes in rhizodeposition. The decrease in plant diversity also reduces the functional diversity of rhizosphere bacteria, weakening plant–microbe interactions. Second, the differences in the quantity and quality of resources returned to the soil by the different plant species have important effects on components of the soil biota and the processes that they regulate (Wardle *et al*., 2004). A significant decline in the number of plant species and changes in dominant species will distinctly alter the connections between plants and microbes, especially the connection between rhizosphere microbes and plants.

The reduction of bacterial and fungal diversity with fertilization could also affect the linkage between plants and microbes. Studies have shown that rhizobia are less able to promote plant growth in soils with N fertilizers (Weese *et al*., 2015). Long‐term fertilization can reduce the richness and diversity of arbuscular mycorrhiza, thus changing the symbiosis between plants and fungi (Corkidi *et al*., 2002; Lin *et al*., 2012).

### Response of soil microbial networks to long‐term fertilization

After 150 years in the long‐term fertilized plot, we found that the complexity of the soil microbial inner network was reduced. Compared with the control plot, the linkages among soil microbes were also reduced whether within areas of 1 m^2^ or 5 m^2^ (Fig. S5 and Table S2). Similarly, fertilization in the first 100 years (from 1870 to 1976) and the latest two decades (from 1984 to 2008) weakened the interaction among soil microbes compared with the control (Fig. S9 and Table S4). This may be due to long‐term fertilization inhibiting certain functions of soil microbes in grassland ecosystems, such as recalcitrant C degradation, N fixation and denitrification. Also, we found that long‐term fertilization significantly reduced the abundance of these functional genes (Fig. 2). This result was similar to Hallin *et al*. (2009), who found 50 years’ ammonium fertilization significantly reduced the abundance of denitrification‐related genes. Since soil microbes are closely linked through their functions and/or roles in the ecosystem, long‐term fertilization may affect the complexity of microbial network by altering their functions. Additionally, we found that the long‐term application of N fertilizer not only increased the N content, but also increased the C content and C/N ratio (Table 1). Considering that a high C/N ratio will not only affect the growth rate of microbes (Bahram *et al*., 2018), but will also affect their utilization efficiency of C and/or N (Mooshammer *et al*., 2014; Geyer *et al*., 2016), we believe that the increased C/N ratio will also affect microbial interactions. This is supported by Morriën *et al*. (2017), who have reported that the C and N utilization efficiency was highly correlated with the nature of microbial networks, such as the clustering coefficient and network density.

In conclusion, we studied how plant communities, microbial functional communities and their networks responded to 150 years of fertilization. Our results showed that inorganic fertilization over more than a century not only reduced the richness and diversity of plants and soil microbes, but also decreased potential associations between plants and functional microbes. Previously, most studies were based on taxonomic and/or phylogenetic diversity but not on functional diversity. It is believed that functional traits could have the most critical impacts on community assembly. The simplification of plant–microbe networks may reduce the capability of plant defence systems to diseases and resilience to biotic and abiotic stresses. Nevertheless, further study of the network characteristics between plants and microbes is needed in order to predict ecosystem function and stability in responses to human long‐term agricultural practices.

## Experimental procedures

### Site and sampling

The Park Grass Experiment (PGE) was established in 1856 on about 2.8 ha of parkland that had been in permanent pasture for at least 100 years previously. The original design included 20 plots with different fertilizer treatments. The original purpose of PGE was to investigate ways of improving the yield of hay by the application of inorganic fertilizers and organic manure. Therefore, every plot was cut each year for hay, usually in June, and a second cut taken in the autumn since 1875. The plots were originally cut by scythe, then by horse‐drawn and then tractor‐drawn mowers. Plant community composition has only been influenced by the different fertilizer treatments since 1857 (Silvertown *et al*., 2006). Detailed harvest methods can be found in e‐RA: the electronic Rothamsted Archive (http://www.era.rothamsted.ac.uk/Park#Harvesting%20methods).

In September 2009, soil samples were taken from the PGE using a spatially explicit nested sampling design in two treatments, of which one has been fertilized with inorganic fertilizers for more than 150 years (11/2C, 18.76 m × 12.57 m), and the other was a control with no fertilizer or lime (12D, l 19.26 m × 12.57 m). Paths (~ 1 m) are maintained between treatments, and plots 11/2C and 12D are completely separate (Fig. S1). Except for fertilization, the management of the two plots was identical at all times. The 11/2C plot has been fertilized with 144 kg N ha^−1^ y^−1^ as ammonium sulfate in spring since 1856 with additional minerals (35 kg P as triple superphosphate, 225 kg K as potassium sulfate, 15 kg Na as sodium sulfate, 10 kg Mg as magnesium sulfate and 450 kg of sodium silicate ha^−1^ y^−1^) applied in winter. All treatments are applied as a top dressing. Originally applications were applied by hand; this is now done by machine. Ground chalk (lime) was applied every third year to maintain soil pH at above 5 in the fertilized (11/2C) plot. At the time of sampling, the pH of the unfertilized plot was 5.1. In September 2009, prior to soil sampling, plant species richness was recorded in each of the nested squares (0.025, 0.1, 0.625, 1, 6.25 and 25 m^2^) in plots 11/2C and 12D. In addition, the smallest quadrat (0.025 m^2^) was also used to record the species immediately above each point where a soil core was taken. Then, a centre and five nested squares around the centre (0.1, 0.25, 1, 2.5 and 5 m^2^) were established on each plot (control and fertilized plots) (Liang *et al*., 2015). Soil samples were collected from the centre and at the four corners of each nested square. A total of 42 soil samples were taken. At each sampling point, a soil core of 2.5 cm diameter and 10 cm depth was taken. Before soil sampling, plant species richness was recorded in each of the nested squares in plots 11/2C and 12D; detailed sampling descriptions can be found in a previous publication (Liang *et al*., 2015).

### Soil analysis, DNA extraction and purification, GeoChip analysis

Soil geochemical properties were measured by Brookside Laboratories, Inc (New Knoxville, OH), including pH, moisture, total N (TN), total C (TC), NO_3_^−^ and NH_4_^+^. DNA extraction and purification were performed as described previously (Zhou *et al*., 1996). GeoChip 3.0 contains ~ 28 000 probes and covers approximately 57 000 gene sequences in 292 functional families (He *et al*., 2010). Hybridization, scanning procedures and instrumental analysis conditions were the same as in our previous study (Liang *et al*., 2015). Spots with signal‐to‐noise ratios lower than 2.0 were removed before statistical analysis as described previously (He *et al*., 2010).

### Statistical analysis

GeoChip data were further analysed with different statistical methods. The GeoChip contains probes for many different types of functional genes, and hence, more broad functional groups can be sampled from a community. Sampling multiple genes of diverse functions could reduce the potential biases from a single gene such as 16S rRNA gene (Zhou *et al*., 2008). Microbial diversity was calculated by Shannon index (Hill *et al*., 2003). CoNet was used to generate interaction networks to determine the effect of 150‐year long‐term fertilization on the intrarelationships among soil microbes, and on the inter‐relationships between aboveground and belowground biological communities. Zero‐rich data were filtered before the network construction. Briefly, the construction of a network graph was divided into four steps: basic configuration, permutation, bootstrapping and restore network from random files. Pairwise associations among species/genes were calculated using the Pearson, Spearman, Bray–Curtis and Kullback–Leibler methods simultaneously. The initial top and bottom edge numbers were set to 2000. An edge‐ and measure‐specific *P*‐value was obtained as the area under the bootstrap distribution limited by the mean of the permutation distribution (Faust and Raes, 2012). The edges were retained when supported by at least two correlation methods. The edges were discarded when the limits of the 95% confidence interval defined by the bootstrap distribution or the adjusted *P*‐values were higher than 0.05. A final network was restored from the permutation and bootstrap files (Wang *et al*., 2017a,b). The graphs of interaction network were built in Cytoscape (version 3.4.0). Each node in the network represents a microbial taxon, and the edges represent the correlation among different nodes. Network density and the clustering coefficient described how well a node was connected with its neighbours (Zhou *et al*., 2011). In addition, temporal series data from the year 1870 to the year 2008 for microbial functional communities were also analysed to determine the effect of fertilization on co‐occurrence networks among soil microbes. Microbial networks were constructed for the two periods: the first one hundred (from 1870 to 1976) and the latest two decades (from 1984 to 2008). Detailed information and descriptions for these data can be found at Liang *et al*. (2019). Mantel test and Pearson's correlation test were performed to determine the effect of soil properties and plant diversity on microbial functional gene composition and structure. The t‐test was used to determine whether there was a significant difference between fertilization and control. Mantel path analysis was used to link the interaction between plants and microbes to several biotic and abiotic attributes (Hartmana *et al*., 2008). All analyses were performed in R (version 3.3.2).

## Conflict of Interest

The authors declare no conflict of interest.

## Author's contribution

All authors contributed intellectual input and assistance to this study and manuscript preparation. J.Z., Y.L., S.M., P.H., I.C., J.S. and L.W. developed the original framework. S.M., P.H., I.C., J.S. and L.W. took the samples. Y.L., R.H. and L.W. contributed to data analysis. Y.L. and L.W. did GeoChip analysis. R.H. and Y.L. wrote the paper with help from S.M., P.H., I.C. and J.S.

## Supporting information


**Fig. S1.** Park Grass Experimental site showing location of sampled areas (red squares, 11/2C and 12/D) within contrasting fertilizer treatments (yellow squares).
**Fig. S2.** Community richness of (a) aboveground plant, (b) all functional genes detected by GeoChip 3.0, (c) bacteria, and (d) fungi in control and long‐term fertilized plots of Park Grass.
**Fig. S3.** The ratio of bacteria to fungi.
**Fig. S4.** The normalized signal intensity of the detected key genes families involved N cycling.
**Fig. S5.** Network interactions among soil microbes in (a, c) control and long‐term (b, d) fertilized plots within (a, b) 1 m^2^ and (c, d) 5 m^2^.
**Fig. S6.** Network interactions between plants and soil microbes in (a) control plots and (b) fertilized plots within a space of 1 m^2^.
**Fig. S7.** Network interactions between plants and soil microbes in (a) control plots and (b) fertilized plots within 5 m^2^.
**Fig. S8.** Linear regression analysis between total carbon (TC) content of soil and total signal intensity of carbon degradation functional genes.
**Fig. S9.** Network interactions among soil microbes at two temporal scales, first one hundred years (1870‐1976) in control (a) and fertilized (b) plots, and latest two decades years (1984‐2008) in control (c) and fertilized (d) plots.

**Table S1.** Pielou evenness and Shannon alpha‐diversity (H′) of plants and all functional genes detected by GeoChip.
**Table S2.** Network topological properties among soil microbes in control and long‐term fertilized plots within areas of 1 m^2^ and 5 m^2^.
**Table S3.** Pearson correlations and *P* values for microbial gene diversity and plant diversity, soil geochemical variables.
**Table S4.** Network topological properties among soil microbes in control and long‐term fertilized plots at two temporal scales, first one hundred years (1870–1976) and latest two decades years (1984–2008).

## Data Availability

GeoChip data are available at Figshare, https://doi.org/10.6084/m9.figshare.7806848. All other relevant data are available from the corresponding author upon request.
