# Supplementary material for: Plant–microbe networks in soil are weakened by century‐long use of inorganic fertilizers
Source: Microb Biotechnol. 2019 Sep 19;12(6):1464–75. doi: 10.1111/1751-7915.13487 (PMC6801139; doi:10.1111/1751-7915.13487)
Supplement: Supplementary file 1 — Fig. S1. Park Grass Experimental site showing location of sampled areas (red squares, 11/2C and 12/D) within contrasting fertilizer treatments (yellow squares). Fig. S2. Community richness of (a) aboveground plant, (b) all functional genes detected by GeoChip 3.0, (c) bacteria, and (d) fungi in control and long‐term fertilized plots of Park Grass. Fig. S3. The ratio of bacteria to fungi. Fig. S4. The normalized signal intensity of the detected key genes families involved N cycling. Fig. S5. Network interactions among soil microbes in (a, c) control and long‐term (b, d) fertilized plots within (a, b) 1 m2 and (c, d) 5 m2. Fig. S6. Network interactions between plants and soil microbes in (a) control plots and (b) fertilized plots within a space of 1 m2. Fig. S7. Network interactions between plants and soil microbes in (a) control plots and (b) fertilized plots within 5 m2. Fig. S8. Linear regression analysis between total carbon (TC) content of soil and total signal intensity of carbon degradation functional genes. Fig. S9. Network interactions among soil microbes at two temporal scales, first one hundred years (1870‐1976) in control (a) and fertilized (b) plots, and latest two decades years (1984‐2008) in control (c) and fertilized (d) plots. [file MBT2-12-1464-s001.docx]

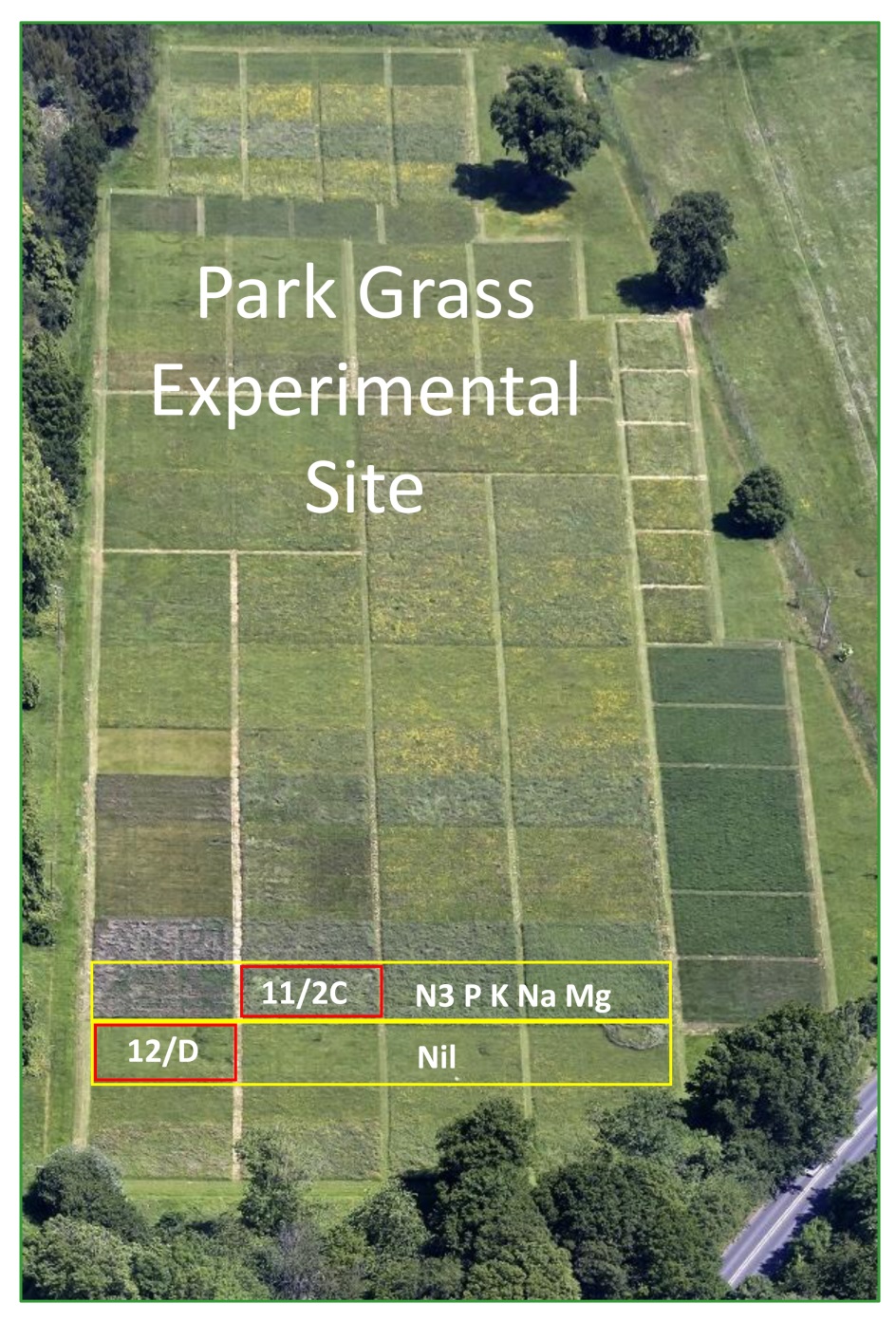


**Fig. S1.** Park Grass Experimental site showing location of sampled areas (red squares, 11/2C and 12/D) within contrasting fertilizer treatments (yellow squares). Paths (3' 4", ~ 1 m) are maintained between treatments; and plots 11/2C and 12D are completely separate with the path between them only at one corner. N3 represents ammonium sulfate supplying 144 kg N and 165 kg S, Nil represents no fertilizer or manure.


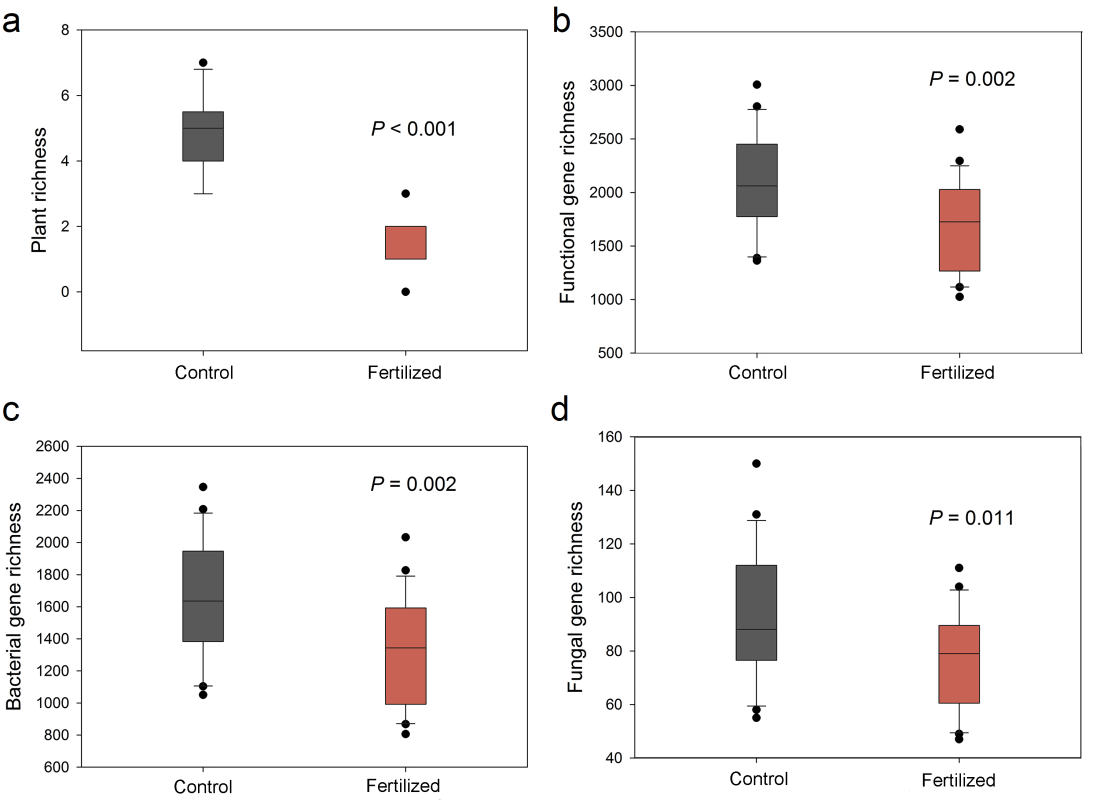


**Fig. S2.** Community richness of (a) aboveground plant, (b) all functional genes detected by GeoChip 3.0, (c) bacteria, and (d) fungi in control and long-term fertilized plots of Park Grass. Significances between the control and fertilized plot were performed by the Student *t*-test.


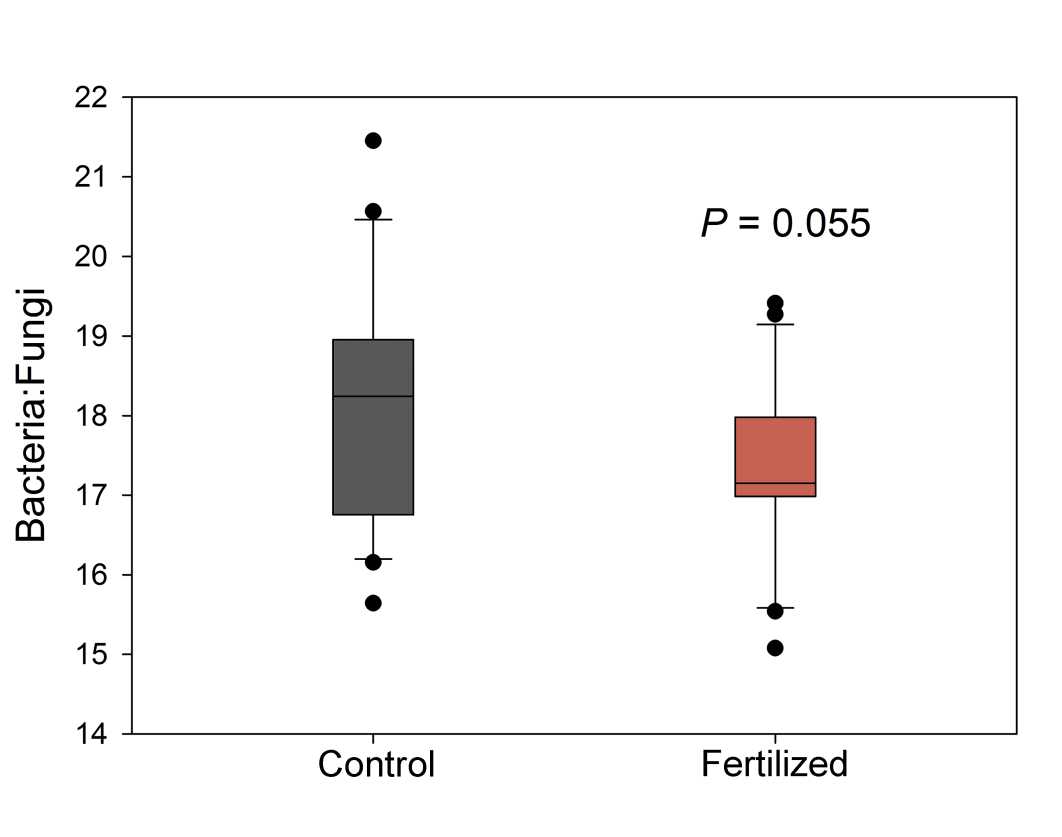


**Fig. S3.** The ratio of bacteria to fungi. Significances between the control and fertilized plot were performed by the Student *t*-test.


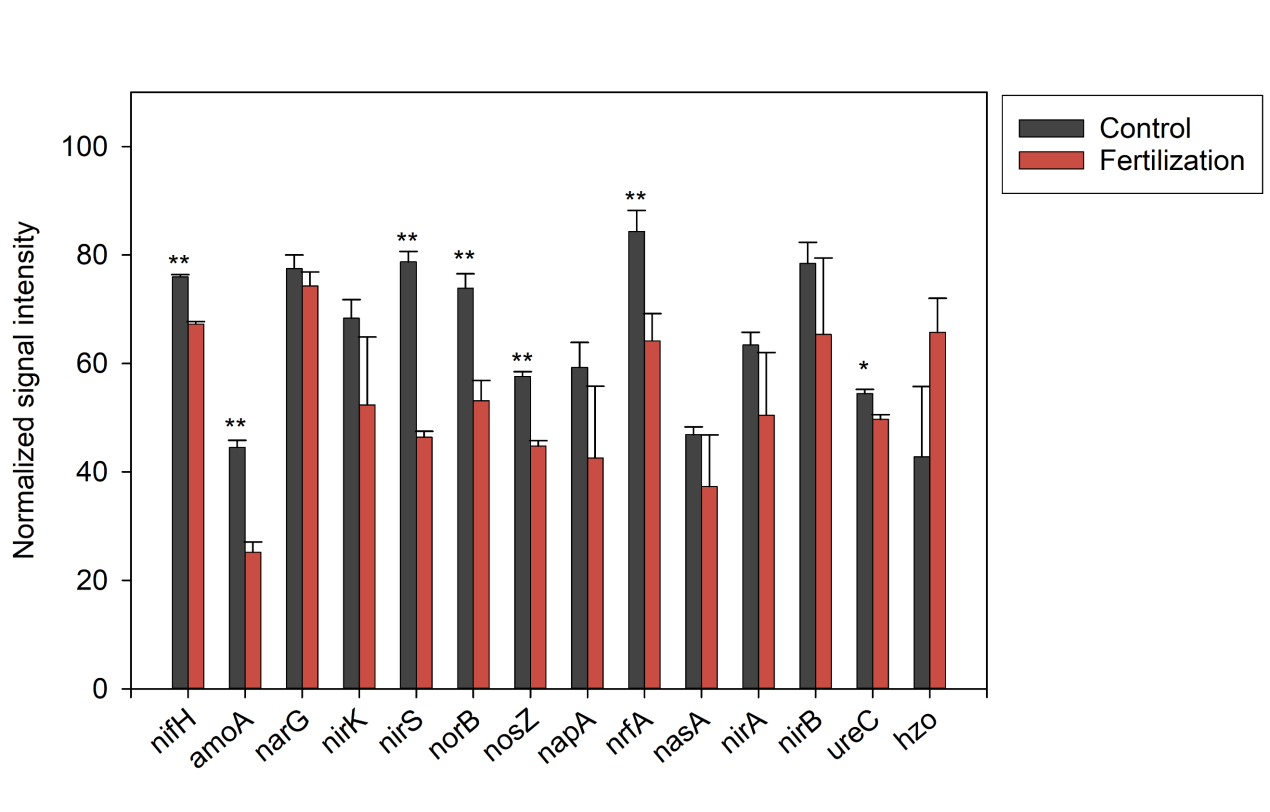


**Fig. S4.** The normalized signal intensity of the detected key genes families involved N cycling. The signal intensities were the sum of detected individual gene sequences for each functional gene. All data are presented as mean ±SD.**p*<0.05, ***p*<0.01.


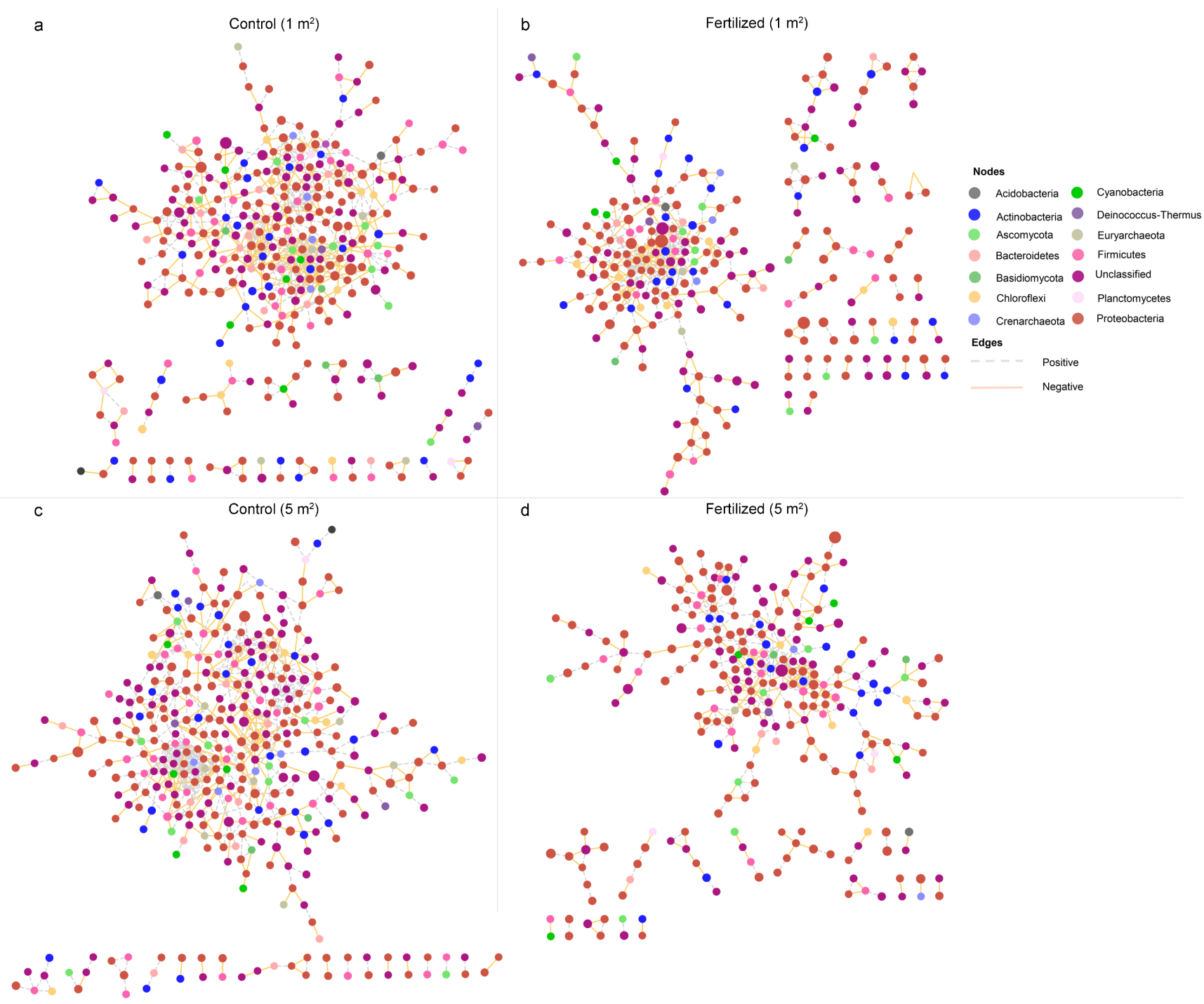


**Fig. S5.** Network interactions among soil microbes in (a, c) control and long-term (b, d) fertilized plots within 1 m^2^ (a, b) and 5 m^2^ (c, d). The node size represents the relative abundance of soil microbes. Dotted grey dash represents positive correlations and orange solid line represents negative correlation.


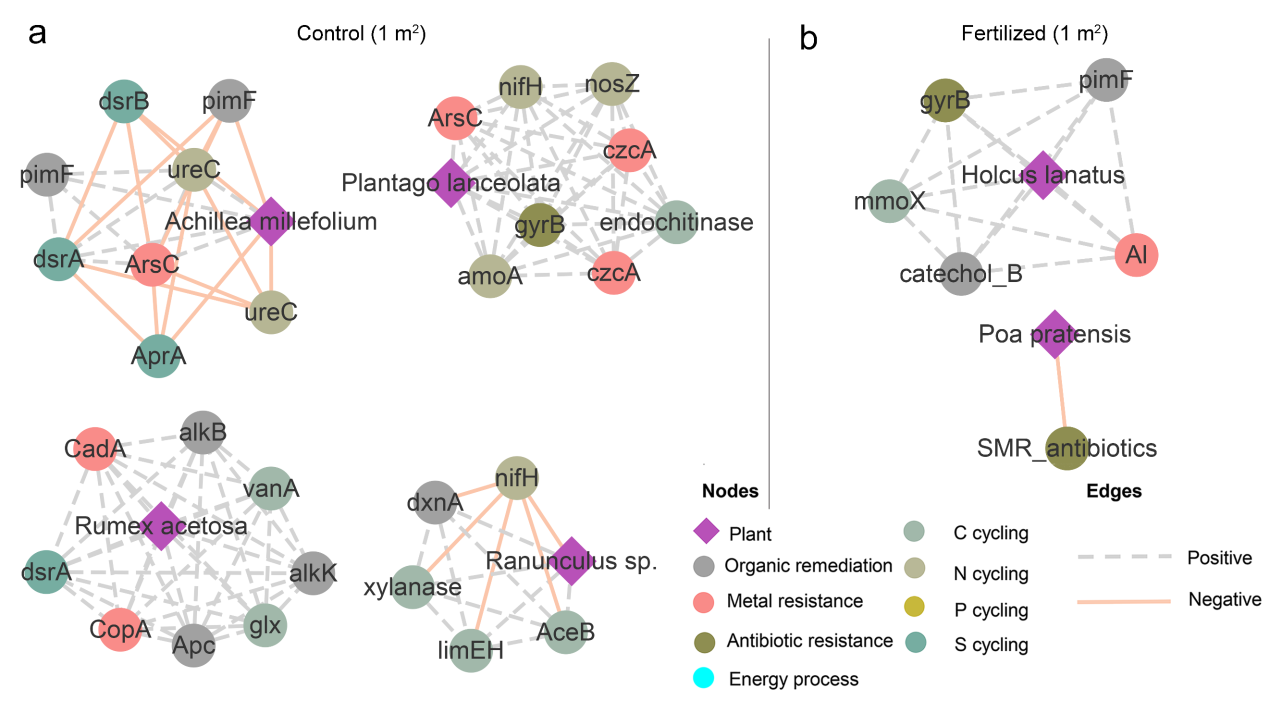


**Fig. S6.** Network interactions between plants and soil microbes in (a) control plots and (b) fertilized plots within a space of 1 m^2^. Circles and diamonds represent functional genes and plants, respectively.


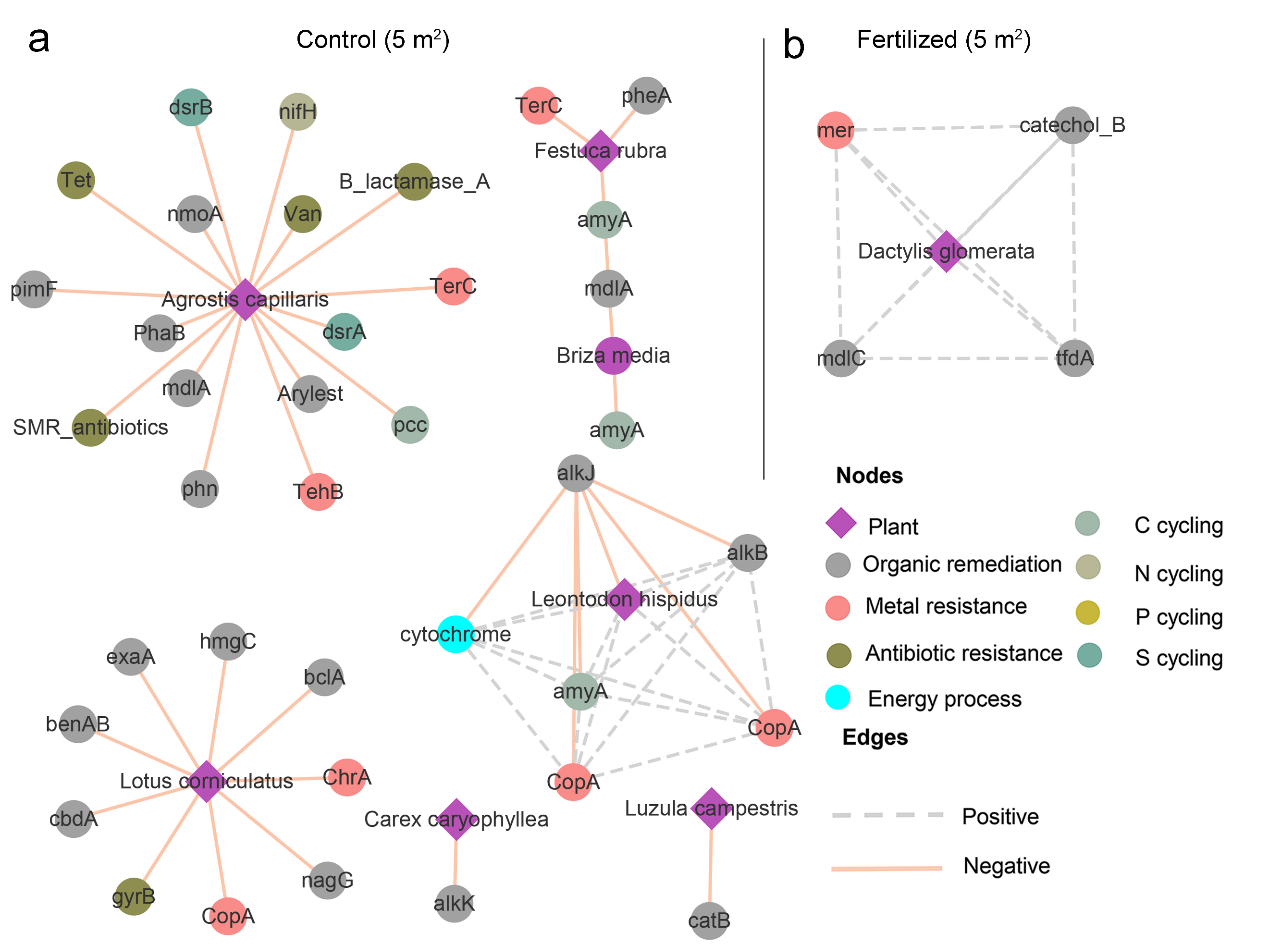


**Fig. S7.** Network interactions between plants and soil microbes in (a) control plots and (b) fertilized plots within 5 m^2^. Circles and diamonds represent functional genes and plants, respectively.


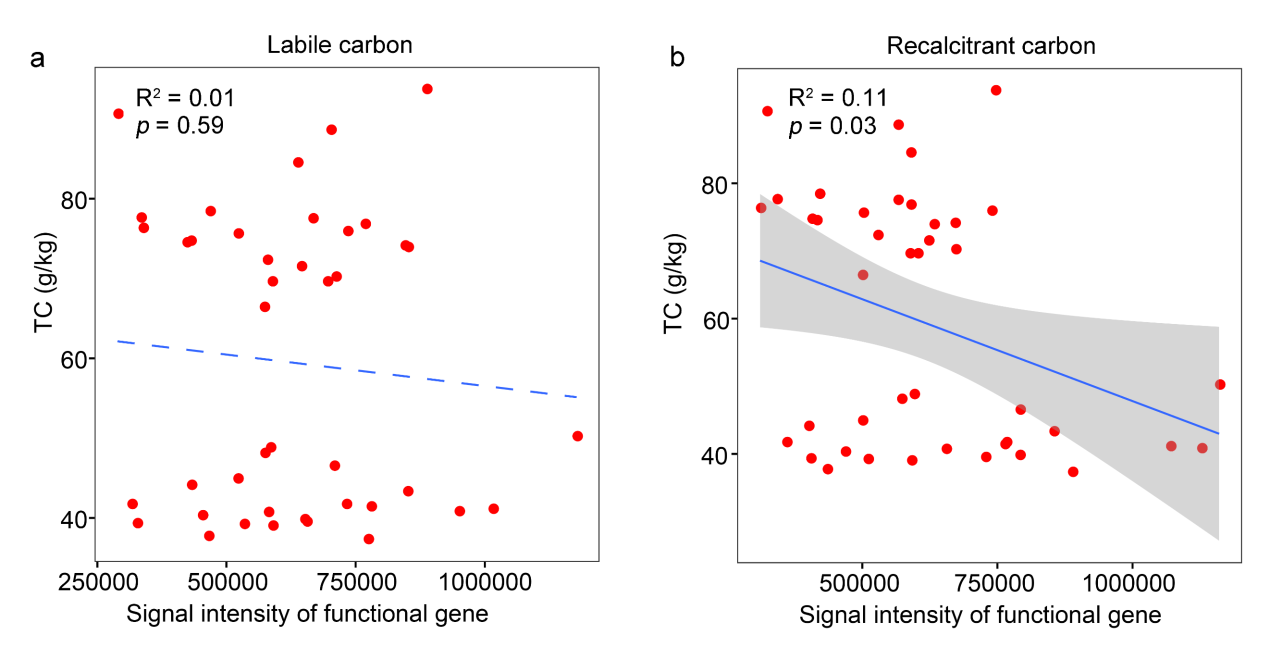


**Fig. S8.** Linear regression analysis between total carbon (TC) content of soil and total signal intensity of carbon degradation functional genes. Labile Carbon included starch, hemicellulose and cellulose; recalcitrant carbon included aromatic carbon, chitin and lignin.


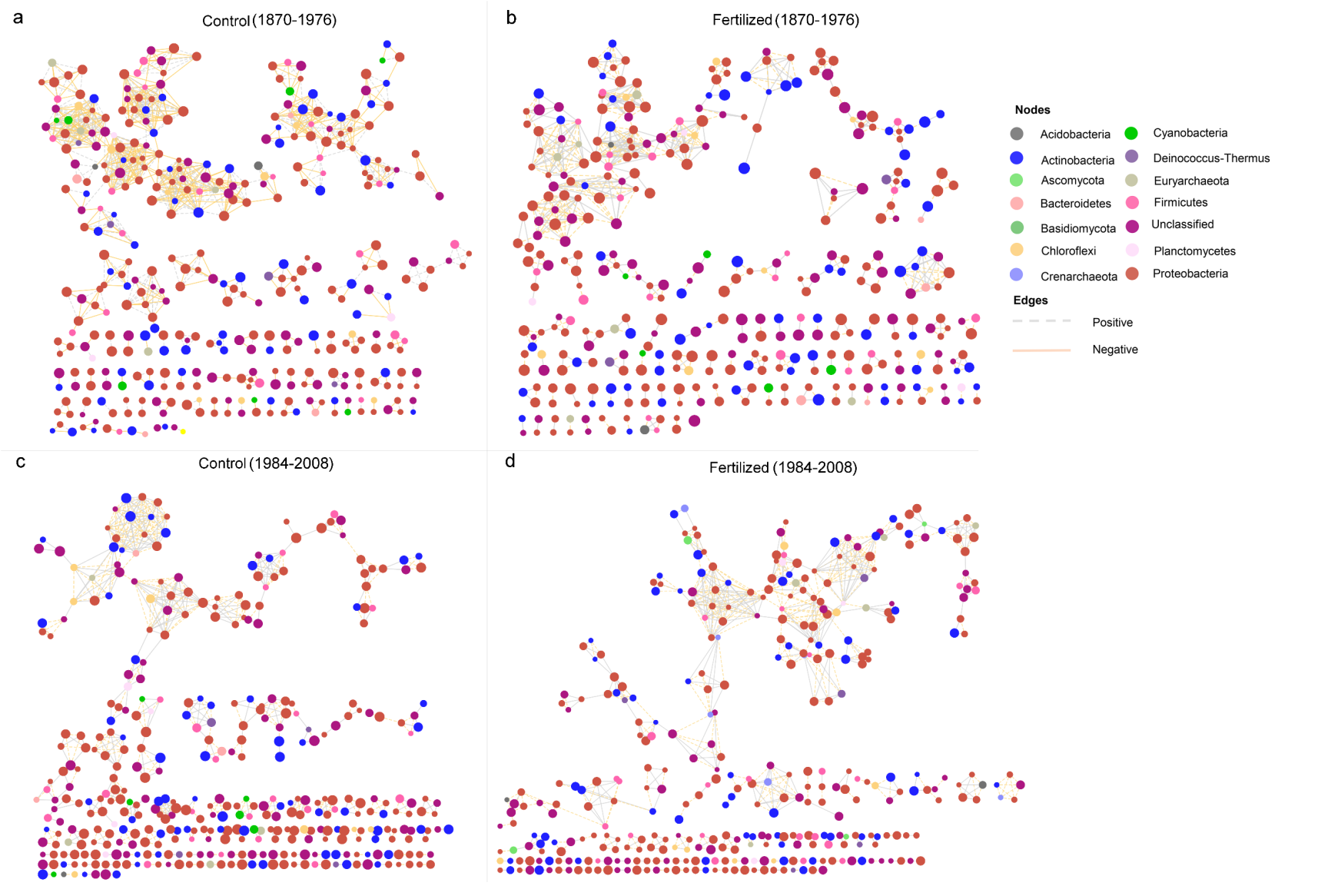


**Fig. S9.** Network interactions among soil microbes at two temporal scales, first one hundred years (1870-1976) in control (a) and fertilized (b) plots, and latest two decades years (1984-2008) in control (c) and fertilized (d) plots. The node size represents the relative abundance of soil microbes. Dotted grey dash represents positive correlations and orange solid line represents negative correlation.
