# Supplementary material for: Plant–microbe networks in soil are weakened by century‐long use of inorganic fertilizers
Source: Microb Biotechnol. 2019 Sep 19;12(6):1464–75. doi: 10.1111/1751-7915.13487 (PMC6801139; doi:10.1111/1751-7915.13487)
Supplement: Supplementary file 2 — Table S1. Pielou evenness and Shannon alpha‐diversity (H′) of plants and all functional genes detected by GeoChip. Table S2. Network topological properties among soil microbes in control and long‐term fertilized plots within areas of 1 m2 and 5 m2. Table S3. Pearson correlations and P values for microbial gene diversity and plant diversity, soil geochemical variables. Table S4. Network topological properties among soil microbes in control and long‐term fertilized plots at two temporal scales, first one hundred years (1870–1976) and latest two decades years (1984–2008). [file MBT2-12-1464-s002.docx]

Table S1 Pielou evenness and Shannon alpha-diversity (H’) of plants and all functional genes detected by GeoChip.

|  | Shannon alpha-diversity (H’) | | | | Pielou evenness | | | |
| --- | --- | --- | --- | --- | --- | --- | --- | --- |
|  | Control | Fertilized | T | *p* | Control | Fertilized | T | *p* |
| Plants | 1.541±0.251 | 0.382±0.038 | 11.588 | **< 0.001** | 4.767±0.881 | 5.061±0.511 | -0.206 | 0.792 |
| All functional genes | 7.389±0.218 | 7.146±0.259 | 3.293 | **0.002** | 0.967±0.002 | 0.967±0.002 | 0.043 | 0.966 |
| Bacterial genes | 7.146±0.224 | 6.899±0.265 | 3.253 | **0.002** | 0.966±0.002 | 0.965±0.002 | 0.288 | 0.775 |
| Fungal genes | 4.283±0.254 | 4.108±0.241 | 2.294 | **0.027** | 0.950±0.006 | 0.956±0.007 | -2.693 | **0.010** |

The significant values (*p* < 0.05) are indicated in boldface. All data are presented as mean ±SD.

Table S2 Network topological properties among soil microbes in control and long-term fertilized plots within areas of 1m^2^ and 5m^2^.

| Networks | Plots | Nodes | Edges | Clustering coefficient | Network diameter | Network density | Avg. number of neighbors |
| --- | --- | --- | --- | --- | --- | --- | --- |
| 1 m^2^ | Control | 359 | 702 | 0.281 | 17 | 0.011 | 3.911 |
|  | Fertilized | 307 | 433 | 0.226 | 23 | 0.009 | 2.821 |
| 5 m^2^ | Control | 386 | 715 | 0.263 | 23 | 0.010 | 3.705 |
|  | Fertilized | 264 | 427 | 0.240 | 20 | 0.012 | 3.235 |

Table S3 Pearson correlations and *p* values for microbial gene diversity and plant diversity, soil geochemical variables.

|  |  | pH | Moisture | TN | TC | C:N | NO_3_^-^ | NH_4_^+^ | Plant diversity |
| --- | --- | --- | --- | --- | --- | --- | --- | --- | --- |
| Plant diversity | r | -0.350^*^ | -0.222 | -0.789^***^ | -0.819^***^ | -0.745^***^ | -0.819^***^ | -0.745^***^ |  |
|  | *p* | 0.023 | 0.157 | <0.001 | <0.001 | <0.001 | <0.001 | <0.001 |  |
| Gene diversity | r | -0.114 | -0.032 | -0.423^**^ | -0.434^**^ | -0.384^*^ | -0.434^**^ | -0.384^*^ | 0.465^**^ |
|  | *p* | 0.474 | 0.842 | 0.005 | 0.004 | 0.012 | 0.004 | 0.012 | 0.002 |
| C gene diversity | r | -0.083 | 0.017 | -0.338^*^ | -0.339^*^ | -0.281 | -0.339^*^ | -0.281 | 0.389^*^ |
|  | *p* | 0.602 | 0.914 | 0.029 | 0.028 | 0.072 | 0.028 | 0.071 | 0.011 |
| N gene diversity | r | -0.138 | -0.043 | -0.426^**^ | -0.431^**^ | -0.364^*^ | -0.431^**^ | -0.364^*^ | 0.485^**^ |
|  | *p* | 0.382 | 0.785 | 0.005 | 0.004 | 0.018 | 0.004 | 0.018 | 0.001 |
| P gene diversity | r | -0.138 | -0.043 | -0.426^**^ | -0.431^**^ | -0.364^*^ | -0.431^**^ | -0.364^*^ | 0.485^**^ |
|  | *p* | 0.382 | 0.785 | 0.005 | 0.004 | 0.018 | 0.004 | 0.018 | 0.001 |

All data are presented as mean ±SD. The symbols ^*^, ^**^, and ^***^ represent *p* < 0.05, *p* < 0.01, and *p* < 0.001, respectively

Table S4 Network topological properties among soil microbes in control and long-term fertilized plots at two temporal scales, first one hundred years (1870-1976) and latest two decades years (1984-2008).

| Networks | Plots | Nodes | Edges | Clustering coefficient | Network diameter | Network density | Avg. number of neighbors |
| --- | --- | --- | --- | --- | --- | --- | --- |
| 1870-1976^†^ | Control | 1604 | 13921 | 0.841 | 33 | 0.011 | 17.358 |
|  | Fertilized | 1603 | 8483 | 0.810 | 32 | 0.007 | 10.584 |
| 1984-2008^†^ | Control | 1618 | 10865 | 0.822 | 26 | 0.008 | 13.430 |
|  | Fertilized | 1604 | 9353 | 0.791 | 37 | 0.007 | 11.662 |
| 1870-1976^§^ | Control | 376 | 1109 | 0.686 | 10 | 0.016 | 5.899 |
|  | Fertilized | 364 | 711 | 0.571 | 14 | 0.011 | 3.907 |
| 1984-2008^§^ | Control | 389 | 718 | 0.678 | 10 | 0.010 | 3.692 |
|  | Fertilized | 366 | 740 | 0.612 | 16 | 0.011 | 4.044 |

^†^ Represents total network in control and long-term fertilized plots in two time periods of 106 and 24 years; ^§^ Functional microbes whose signal intensity accounted for more than 0.05% of the total signal intensity were selected from the total network to show the linkages among microbes.
